# MOFs-Derived Porous NiFe_2_O_4_ Nano-Octahedrons with Hollow Interiors for an Excellent Toluene Gas Sensor

**DOI:** 10.3390/nano9081059

**Published:** 2019-07-24

**Authors:** Yanlin Zhang, Chaowei Jia, Qiuyue Wang, Quan Kong, Gang Chen, Hongtao Guan, Chengjun Dong

**Affiliations:** 1 School of Materials Science and Engineering, Yunnan University, Kunming 650091, China; 2 Yunnan Province Key Lab of Micro-Nano Materials and Technology, Yunnan University, Kunming 650091, China

**Keywords:** MOFs, NiFe_2_O_4_, octahedron, gas sensor, toluene

## Abstract

Toluene is extensively used in many industrial products, which needs to be effectively detected by sensitive gas sensors even at low-ppm-level concentrations. Here, NiFe_2_O_4_ nano-octahedrons were calcinated from NiFe-bimetallic metal-organic framework (MOFs) octahedrons synthesized by a facile refluxing method. The co-existence of p-Phthalic acid (PTA) and 3,3-diaminobenzidine (DAB) promotes the formation of smooth NiFe-bimetallic MOFs octahedrons. After subsequent thermal treatment, a big weight loss (about 85%) transformed NiFe_2_O_4_ nanoparticles (30 nm) into NiFe_2_O_4_ porous nano-octahedrons with hollow interiors. The NiFe_2_O_4_ nano-octahedron based sensor exhibited excellent gas sensing properties for toluene with a nice stability, fast response, and recovery time (25 s/40 s to 100 ppm toluene), and a lower detection limitation (1 ppm) at 260 °C. The excellent toluene-sensing properties can not only be derived from the hollow interiors combined with porous nano-octahedrons to favor the diffusion of gas molecules, but also from the efficient catalytic activity of NiFe_2_O_4_ nanoparticles.

## 1. Introduction

Toluene is one kind of a colorless substance with a unique aromatic odor that is widely used in commercial products and industrial applications. Unfortunately, toluene is seriously harmful to human health as it strongly affects the nervous system to induce brain function disturbances and damage to the kidneys or liver [1,2]. In addition, toluene is considered as one of the important biomarkers for a diagnosis of lung cancer [3,4]. Moreover, toluene is easily flammable when the temperature is above 4.4 °C [5,6]. Due to the harmful effects of toluene, it is of great urgency to be able to effectively detect toluene. To date, various gas sensors have been developed. In particular, resistive-based gas sensors on a metal oxide base are the most attractive with respect to their cost-effective, versatile, and simple fabrication techniques. Recently, p-type oxide semiconductors have been used as sensing materials due to their good catalysts and multivalent characteristics that favor the selective oxidation of various volatile organic compounds (VOCs) [7]. Although many p-type oxide semiconductors such as NiO [8], CuO [9], Co_3_O_4_ [10], Cr_2_O_3_ [11], Mn_3_O_4_ [12], and so on have been reported to have potential for gas sensors, it is still far less than their counterpart, n-type oxide semiconductors.

Recently, spinel ferrites, MFe_2_O_4_ (M = Zn, Ni, Co, Cu, Mn, Cd, etc.), which are materials that include two types of transition metals, have stood out due to their robust catalytic activity as exhibiting significant potential applications in various fields. In particular, a few of them such as ZnFe_2_O_4_ [13,14], NiFe_2_O_4_ [15,16,17,18,19,20,21,22], CoFe_2_O_4_ [23], MnFe_2_O_4_ [24], Ni_1-X_Co_X_Fe_2_O_4_ [25], and NiCuZn ferrites [26] have been reported to be greatly promising in the electrical properties of gas sensors and humidity sensors. More interesting, NiFe_2_O_4_ with various microstructures have been prepared so far to effectively detect acetone [14,15,16,17], ammonia [18], NO_2_ [19], n-propanol [20], and triethylamine [21]. However, intense efforts are expected to further enhance the sensing properties of the NiFe_2_O_4_ based gas sensor. Designing NiFe_2_O_4_ with unique microstructures is considered to be the most highly promising way. To date, templates (hard template, soft template, and bio-template) are widely used to tune the morphologies of products [27,28,29]. In particular, soft templates are beneficial for the synthesis of different materials to tune the morphologies under relatively mild experimental conditions. Typically, self-assembly occurs involving the soft template, precursor, and solvent molecules. Organic ligands can be used as a sufficient soft template to coordinate metal ions to obtain a unique structure. For instance, Prussian blue analogue derived porous NiFe_2_O_4_ nanocubes have been synthesized, which exhibit sensitivity and selectivity to acetone at low operating temperatures [16].

Today, MOFs have attracted particular attention as sacrificial templates to obtain designable nanoarchitectures [30] with intriguing properties aside from their direct applications in energy storage [31], catalysis [32], electronic devices [33], drug delivery [34], adsorbents [35], separation [36], etc. Among uncountable MOFs, Fe-containing MOFs have been extensively studied thus far due to their good diversity, low toxicity, excellent stability, and easy functionality [37]. Taking the template of MIL-88-Fe, spindle like α-Fe_2_O_3_ with porous structures have been reported to enhance electrochemical performance [38]. In addition, the NiFe bimetallic MOFs have been synthesized by metal Ni incorporation, which is further converted to ternary oxides of NiFe_2_O_4_. Song et al. obtained hollow NiFe_2_O_4_ microspindles from NiFe bimetallic MOFs, illustrating a high sensitivity for acetone detection [14]. Zhang et al. prepared 1D α-Fe_2_O_3_/NiFe_2_O_4_ heterojunction, which showed an improved response to acetone vapors using NiFe-bimetallic MOFs [39]. Previously, we prepared 1D porous NiFe_2_O_4_ nanorods using 2-aminoterephthalic acid as the ligand, which showed excellent selectivity and sensitivity to toluene [40]. Nevertheless, as of now, tuning the NiFe_2_O_4_ microstructures on a NiFe-bimetallic MOFs template base is still developing and in the early stage.

As discussed above, we herein combined both PTA and DAB to prepare NiFe-bimetallic MOFs octahedrons. After thermal treatment, porous NiFe_2_O_4_ nano-octahedrons with hollow interiors were obtained by assembling a large number of nanoparticles. The gas sensor based on NiFe_2_O_4_ nano-octahedrons showed excellent gas sensing properties for toluene detection at low-ppm-levels with good reproducibility, a fast response and recovery time, and a lower detection limit at the optimum operating temperature. Thus, the gas sensing mechanism for the NiFe_2_O_4_ based sensor is understood in detail.

## 2. Materials and Methods

### 2.1. Materials Synthesis

**Figure 1:**
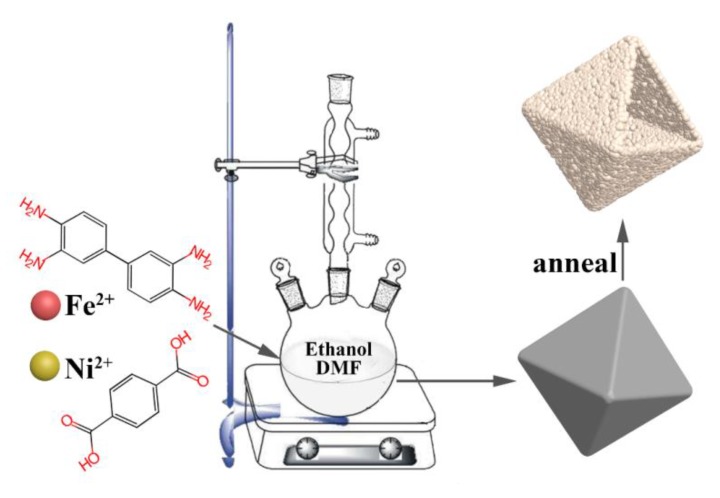
Schematic illustration of the fabrication of the NiFe_2_O_4_ nano-octahedrons.

All the starting reagents were of analytical grade (AR grade) and unpurified. Highly pure water (18 MΩ cm at 25 °C) was used throughout the experiments. First, the octahedron NiFe-bimetallic MOFs were synthesized through a refluxing method, as schematically shown in Figure 1, which were further transferred into NiFe_2_O_4_ with thermal treatment. Typically, 9 mmol of p-Phthalic acid (PTA) was completely dissolved in 50 mL *N*,*N*-Dimethylformamide (DMF). At the same time, 0.3 mmol 3,3-diaminobenzidine (DAB) was dissolved into 30 mL ethanol. Next, the ethanol was slowly added to form a homogenous solution. When the desirable NiCl_2_·6H_2_O (0.5 mmol) and FeCl_2_·4H_2_O (1 mmol) were dissolved into 10 mL DMF, it was dropped into the above mixture with vigorous stirring for 30 min. The whole mixture was refluxed at 120 °C by an oil bath under magnetic stirring for 3 h. Subsequently, the NiFe-bimetallic MOFs were collected after washing several times with ethanol and deionized water, and then air dried at 60 °C for 24 h. Finally, the as-prepared NiFe-precursor was thermally treated at 350 °C for 1 h, and then at 500 °C for a further hour in air at a heating rate of 3 °C/min. Therefore, the NiFe_2_O_4_ nano-octahedrons were obtained. 

### 2.2. Materials Characterization

The structure of NiFe_2_O_4_ was characterized using a Rigaku TTRIII x-ray diffraction with Cu K_α_ radiation (1.540 Å). A FEI QUANTA 200 microscope was used to investigate the morphology of NiFe_2_O_4_ and the NiFe-precursor. The TEM and HRTEM images and corresponding elemental mapping images were obtained by a JEOL JEM-2100 microscope. X-ray photoelectron spectroscopy (XPS) spectra were also measured under Al *K*_α_ X-ray radiation at 15 kV, which were calibrated by a C 1s peak (284.6 eV). An American TA SDT-2960 thermal analyzer was used to examine the transformation from the NiFe-precursor to the final NiFe_2_O_4_ (heating rate of 10 °C/min). After degassing at 300 °C for 3 h under vacuum, a Micromeritics ASAP 2010 automated sorption analyzer was applied to carry out the nitrogen adsorption/desorption measurements.

### 2.3. Fabrication and Measurement of the Gas Sensor

Following previous works, the gas sensor was fabricated and tested [41,42]. The slurry was formed by dispersing the NiFe_2_O_4_ powder in proper deionized water, which was coated on the surface of an alumina tube. In order to form a resistive-based gas sensor, a pair of Au electrodes were passed at each end of the tube, which was further connected by Pt wires. In general, the thickness of the sensing film was about 0.6–0.8 mm. After drying at 120 °C for 2 h, the sensor was annealed at 400 °C for 2 h in air. Then, a Bakelite base was connected to the Pt wires and a Ni–Cr alloy wire was inserted into the tube to perform measurements in a WS-30A system. During the test, the dry air was taken as the reference to mix with an evaporated target solution to obtain the desired concentration. As a typical p-type material, the response (β) of the sensor is generally defined as *R*_g_/*R*_a_, (*R*_a_ and *R*_g_ represent the resistance of the sensor in air and in the presence of target gases, respectively). Furthermore, the response and recovery time were evaluated by the time for 90% of the initial equilibrium resistance change in the adsorption and desorption processes.

## 3. Results

**Figure 2:**
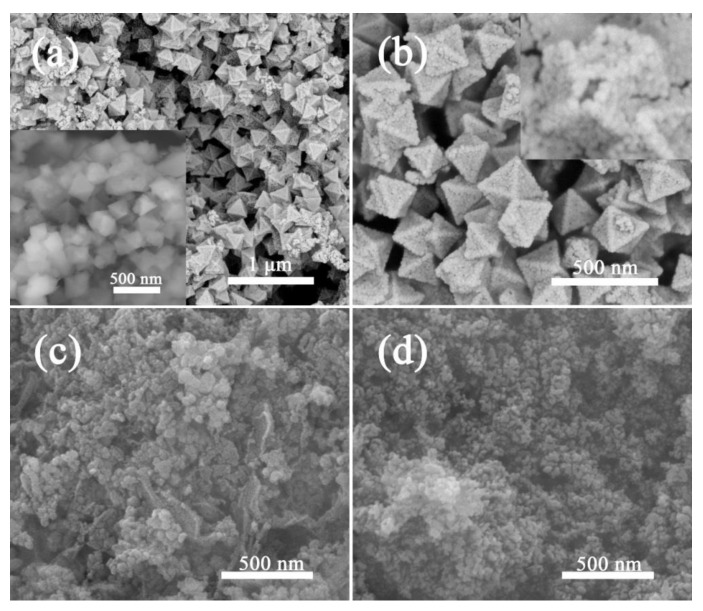
FESEM images of NiFe_2_O_4_ nano-octahedrons in low (**a**) and high magnification (**b**), and NiFe_2_O_4_ synthesized without DAB (**c**), or PTA (**d**). Insets show the FESEM images of NiFe-bimetallic MOFs (**a**) and the hollow interior of NiFe_2_O_4_ (**b**).

NiFe-bimetallic MOFs octahedrons were synthesized by a simple refluxing method, as schematically shown in Figure 1, which was used as the self-sacrificial template for NiFe_2_O_4_ fabrication. It is clear that uniform NiFe-MOFs octahedrons were well-defined with an average size of about 300 nm, as shown in the inset of Figure 2a. Close observation revealed that these NiFe-MOFs octahedrons were smooth on the surface. The as-prepared NiFe-MOFs octahedrons were further thermally transformed into NiFe_2_O_4_ nano-octahedrons in an air atmosphere at a heating rate of 3 °C/min. After thermal treatment, it can be seen that the configuration of the NiFe-MOFs octahedrons was perfectly preserved, as shown in Figure 2a,b. However, the NiFe_2_O_4_ nano-octahedrons were assembled by a large number of nanoparticles, leading to a rough surface. Importantly, the hollow interiors were strongly confirmed by a broken NiFe_2_O_4_ nano-octahedron in the inset of Figure 2b.

**Figure 3:**
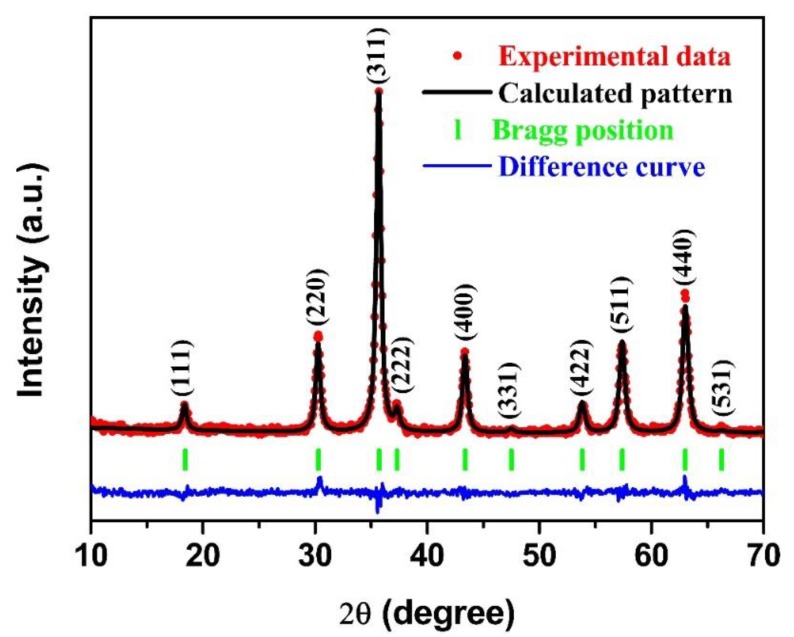
XRD patterns for the experimental and Rietveld refinement data for the NiFe_2_O_4_ nano-octahedrons.

The crystal structure of the NiFe_2_O_4_ nano-octahedrons was studied by XRD measurements, as shown in Figure 3. Compared with the JCPDS: 54-0964, as indicated by the Bragg position at the bottom of the figure, a pure specimen was identified with a cubic NiFe_2_O_4_ structure (space-group: $Fd\bar{3}m$). The appearance of no other diffraction peaks confirmed the high phase purity. The structural parameters were further analyzed by Rietveld refinement based on the Maud program [43]. The difference curve was not greatly fluctuated, which is a sign that the calculated pattern had good consistence with the experimental data in the whole recorded angles from 10–70°. The lattice parameters of a = 8.341092 Å were obtained. Moreover, the crystallite size of around 25.56 nm was estimated. These strong and narrow diffraction XRD peaks are persuasive evidence that an individual NiFe_2_O_4_ phase was completely crystallized by the above thermal treatment of NiFe-bimetallic MOFs.

**Figure 4:**
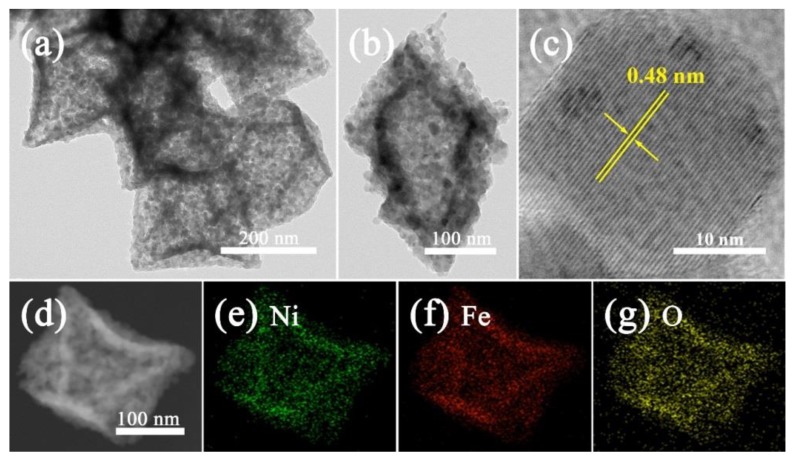
TEM (**a**,**b**) and HRTEM (**c**) images of the NiFe_2_O_4_ nano-octahedrons and elemental mapping images of Ni (**e**), Fe (**f**), and O (**g**) for an individual NiFe_2_O_4_ nano-octahedron (**d**).

**Figure 5:**
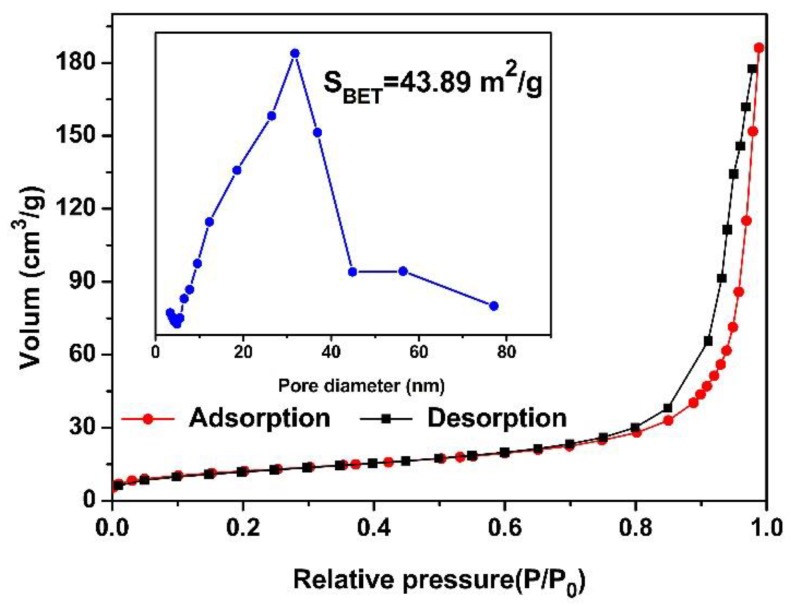
Nitrogen adsorption–desorption isotherm along with the pore size distribution of the NiFe_2_O_4_ nano-octahedrons.

In order to gain further insight into the structural details on NiFe_2_O_4_ nano-octahedrons, TEM and HRTEM measurements were carried out. The TEM observations in Figure 4a demonstrate that a large number of ultrafine nanoparticles (an average diameter of about 30 nm) were homogeneously distributed within the interconnected walls of the hollow octahedrons. From a featured octahedron in Figure 4b, the hollow interior was clearly distinguished by the dark boundary, indicating that the hollow interior was formed by porous walls of around 50 nm in thickness. The lattice fringes of 0.48 nm in the HRTEM image (Figure 4c) could be assigned to the (111) interplane spacing of NiFe_2_O_4_. Moreover, the porous structure and the specific surface area of NiFe_2_O_4_ was investigated using the nitrogen adsorption–desorption isotherms, as shown in Figure 5. Clearly, the sample showed an obvious H3 hysteresis loop, which can be described as a type IV isotherm, implying the presence of a mesoporous and hollow structure. The pore size distribution of the NiFe_2_O_4_ nano-octahedrons calculated using the BJH method was centered at 31.78 nm, as shown in the inset of Figure 5. A BET surface area of 43.89 m^2^/g was obtained. This should offer our NiFe_2_O_4_ a sufficient interface and porous structure to facilitate the gas sensing properties. Furthermore, EDS elemental mapping images of individual NiFe_2_O_4_ nano-octahedrons (Figure 4d–g) demonstrate the homogeneous coexistence of Ni, Fe, and O elements.

**Figure 6:**
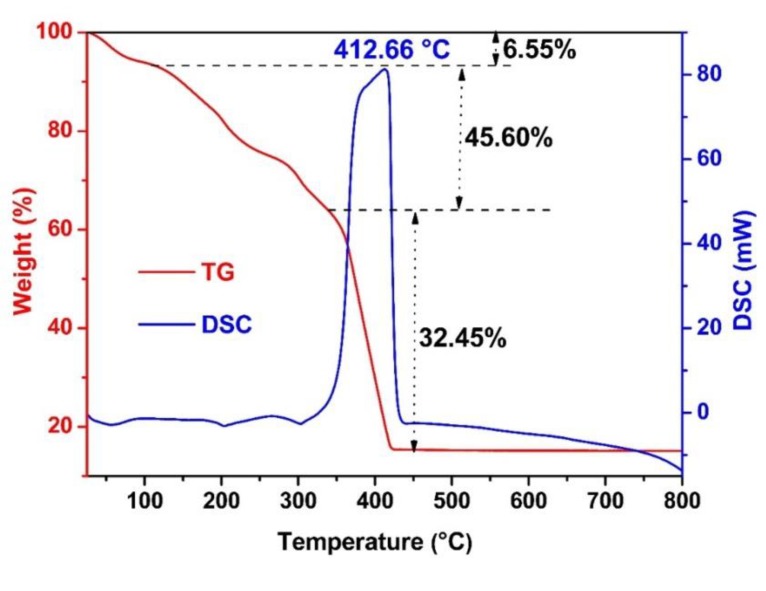
TGA–DSC curves of transformation from NiFe-bimetallic MOFs to NiFe_2_O_4_.

Thermogravimetric measurements were performed to explore the transformation from NiFe- bimetallic MOFs to NiFe_2_O_4_. From the TG curve shown in Figure 6, it can be seen that about 6.55% weight loss was observed in the temperature blow 120 °C, which originated from the loss of absorbed water in the precursor. By further increasing the temperature to 350 °C, around 45.60% weight loss occurred due to the decomposition of organic ligands in the NiFe-bimetallic MOFs. Up to 425 °C, 15% weight was maintained. Accordingly, a clear exothermal peak at 412.66 °C in the DSC curve was observed, suggesting the transformation of NiFe-bimetallic MOFs to NiFe_2_O_4_. It is noteworthy that the large weight loss can be reasonably explained by the large numbers of organic ligands in the NiFe-bimetallic MOFs synthesized by coupling DAB with PTA in a mixed solution of DMF and ethanol.

Taken together, the porous NiFe_2_O_4_ nano-octahedrons were thermally obtained from NiFe-bimetallic MOFs synthesized by a facile refluxing route, which was assembled by a large number of nanoparticles. The existence of abundant organic ligands was revealed by the large weight loss in TG measurements due to the cooperative effects of PTA and DAB in the mixture of DMF and ethanol. It has been reported that Schiff-base polymer spheres have been obtained using certain amounts of PTA and DAB in ethanol at 80 °C under stirring-reflux, which can be further transformed into N-doped porous carbons [44]. To understand the growth mechanism, contrast experiments were conducted only dependent on PTA or DAB following the same experimental process, respectively. Without DAB, the NiFe_2_O_4_ nanoparticles were observed with some sheets, as shown in Figure 2c. In the presence of DAB, the NiFe_2_O_4_ nanoparticles seemed to be more uniform (Figure 2d). Therefore, the combination of PTA and DAB plays a critical role in coordinating with the metal ions to produce octahedral NiFe-bimetallic MOFs through the refluxing approach.

**Figure 7:**
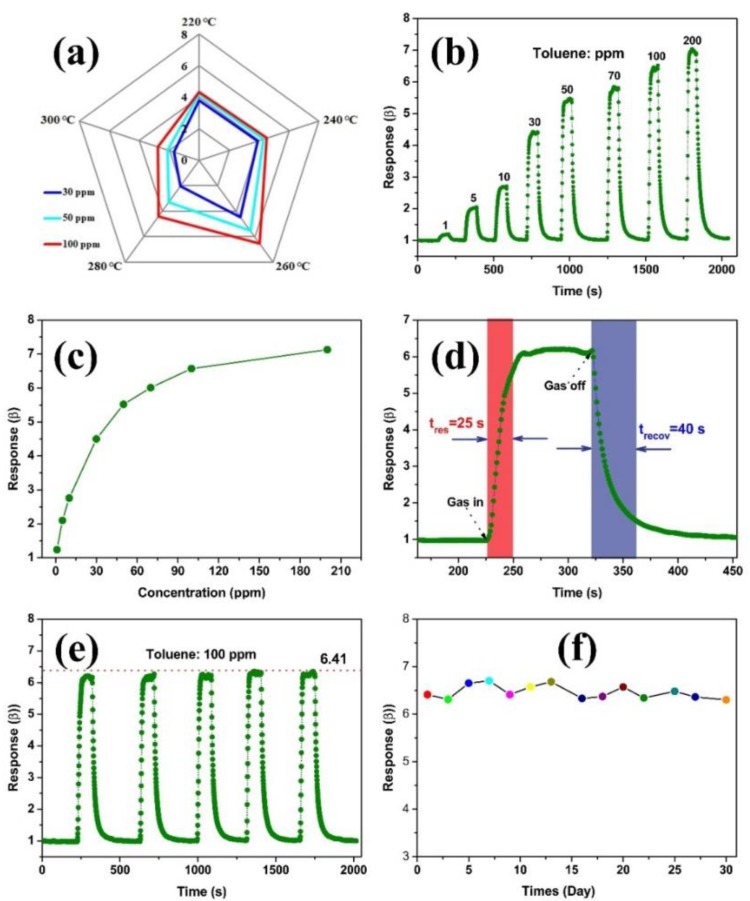
Polar graphs shows the gas responses of gas sensors based on NiFe_2_O_4_ nano-octahedrons as 30, 50, and 100 ppm toluene vs. operating temperatures from 220–300 °C (**a**). Dynamic sensing plot of sensors based on NiFe_2_O_4_ nano-octahedrons upon exposure to toluene (**b**). Concentration dependent response curve vs. the toluene concentrations (1–200 ppm) (**c**). Response/recovery graph to 100 ppm toluene (**d**), and reproducibility of sensor upon exposure (five cycles) to 100 ppm toluene at 260 °C (**e**). Long-term stability (30 days) of sensor to 100 ppm toluene (**f**).

To evaluate the potential practicality and reliability of the gas sensor based on NiFe_2_O_4_ nano-octahedrons, the fundamental properties of a gas sensor were thoroughly studied. The responses of our NiFe_2_O_4_ based sensor to 100 ppm toluene at various operating temperatures from 220–300 °C are shown in Figure 7a. Clearly, the responses kept increasing to reach a maximum at 260 °C and then started to decrease with further increased temperatures. At lower temperatures, the slow diffusion of the tested gas molecules and the insufficient thermal energy to accelerate the reaction between the gas molecules and adsorbed oxygen species is believed to result in a relatively low response. The existence of oxygen species will be given in the XPS analysis below. Although enough thermal energy is available at higher temperatures, it could reduce the gas adsorption capacity from the sensing materials, leading to a decreased response [45,46]. Hence, the gas sensing measurements in the following were performed at 260 °C.

Figure 7b shows the dynamic responses of the NiFe_2_O_4_ nano-octahedrons to 1–200 ppm toluene. It can be seen that a stepwise distribution of the curves appeared by switching dry air and a certain concentration of the target gas. With increasing toluene concentrations, the responses of the sensor rapidly increased. Specifically, the sensor showed approximately responses of 1.24, 2.10, 2.76, 4.50, 5.52, 6.01, 6.57, and 7.13 to 1, 5, 10, 30, 50, 70, 100, and 200 ppm of toluene, respectively, as shown in Figure 7b. It is noteworthy that the responses sharply increased at lower toluene concentrations because the coverage of the gas molecules was low on the surface of the sensing material (Figure 7c). In our case, the sensor was capable of detecting 1 ppm toluene. With the further increase in the toluene concentration, the material surface was gradually covered completely. Thus, the rising trend of responses gradually slowed down, indicating the saturation of the sensor to some extent [47]. Taking 100 ppm toluene as an example, the sensor exhibited fast response/recovery times of 25 s/40 s (Figure 7d). In addition, the responses were almost maintained for the five cyclic toluene exposures, as displayed in Figure 7e, which showed good repeatability within a fluctuation of 2.2%. Furthermore, the long-term stability of the gas sensor was evaluated. The continuous tests were carried out over 30 days with a one day interval while exposing the sensor to 100 ppm toluene. As shown in Figure 7f, the sensor exhibited small deviations (6.2%) during the test over 30 days, demonstrating the good stability of the NiFe_2_O_4_. Importantly, our NiFe_2_O_4_ based gas sensor showed a comparable sensing performance with typical p-type nanomaterials, as summarized in Table 1.

**Figure 8:**
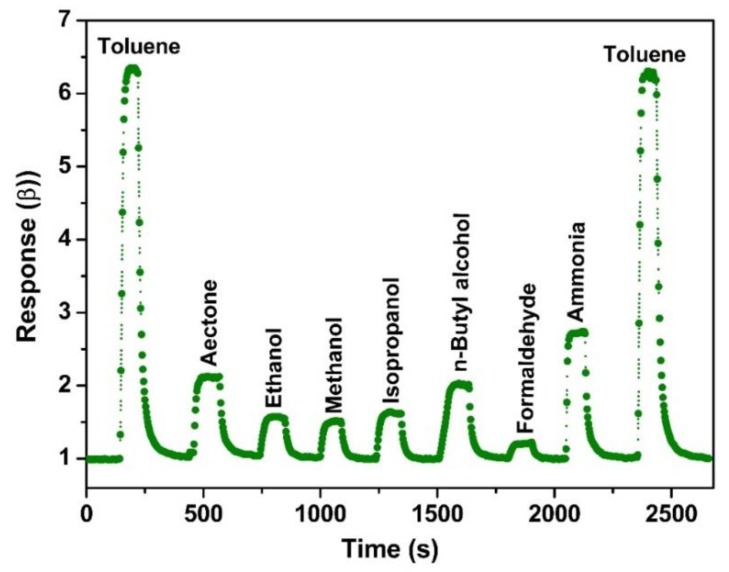
Selectivity of NiFe_2_O_4_ nano-octahedrons to 100 ppm of various gases at 260 °C.

The selectivity generally demonstrated the capability that the gas sensor had to distinguish the target gas without any interference, which is also important for practical applications. Figure 8 compares the sensing response of the NiFe_2_O_4_ nano-octahedron based sensor to 100 ppm toluene, acetone, ethanol, methanol, isopropanol, n-butyl alcohol, formaldehyde, ammonia, and toluene at the same operating temperature of 260 °C. Against the interferences, the gas sensor showed a relatively selective and sensitive detection toward toluene. One plausible explanation for the selective detection of toluene could be ascribed to the excellent catalytic nature of p-type NiFe_2_O_4_ to toluene, leading to more released electrons back to the NiFe_2_O_4_ materials after the reactions of target molecules on the surface [58,59]. Moreover, the optimum operating temperature of 260 °C could provide suitable active energy to promote the reactions of toluene on the NiFe_2_O_4_ surface. Thus, a big change of resistance for the sensor occurred, which exhibits excellent sensing performances.

**Figure 9:**
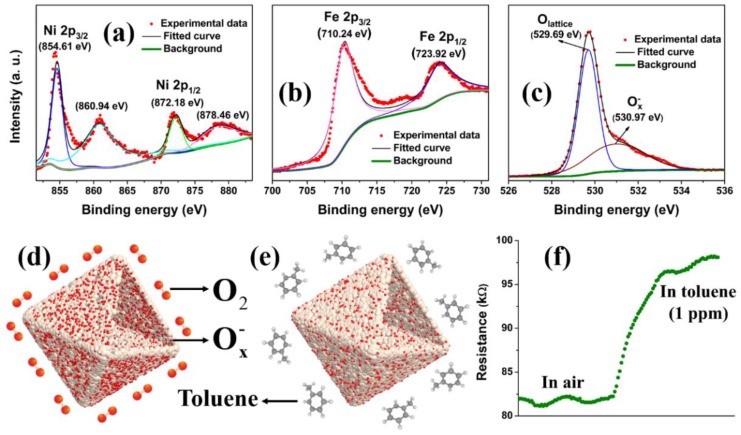
High-resolution XPS spectra for Ni 2p (**a**), Fe 2p (**b**), and O 1s (**c**), and a schematic illustration of the sensing mechanism of the NiFe_2_O_4_ nano-octahedrons in air (**d**), and in toluene (**e**) with the obverse real resistance change in air and 1 ppm toluene (**f**).

As defined above, the sensing response of a semiconductor is dependent on the variation of resistance, which is highly related to both the surface compositions and valance states of the elements in the sensing materials. Therefore, the XPS investigation was conducted, as shown in Figure 9. The high resolution XPS analysis of the Ni 2p spectra in Figure 9a displays the specific peaks of Ni 2p_1/2_ and Ni 2P_3/2_ at the binding energies of 8723.18 eV and 854.61 eV with two apparent satellite peaks at 878.46 eV and 860.94 eV, respectively. Meanwhile, the Fe-related fitting peaks with binding energy values of 723.92 eV and 710.24 eV, corresponded well with Fe 2p_1/2_ and Fe 2p_3/2_, respectively (Figure 9b). The XPS results verify the Ni(II) and Fe(III) in the NiFe_2_O_4_ [39]. In particular, the O 1s XPS spectrum, as shown in Figure 9c, could be resolved by two peaks located at 529.69 eV and 530.97 eV, implying various oxygen contributions. Specifically, the peak at the low binding energy of 529.69 eV is typically assigned to that of lattice oxygen (O_lattice_), which is unreactive with target gases. In contrast, the well-resolved peak at a higher binding energy of 530.97 eV was assigned to surface adsorbed oxygen species, which evolved into O_2_^−^, O^−^, and O^2−^. These oxygen species are believed to play a critical role in reacting with the target gas to determine the gas sensing properties [60]. As is well known, O_2_ has a stronger electron affinity, making it adsorbed on the surface of NiFe_2_O_4_ even at room temperature though a physical process.

When the operating temperature rose to 260 °C, a more stable adsorbed oxygen species can be formed. Thus, the mechanism governing the sensing properties of NiFe_2_O_4_ nano-octahedrons can be systematically discussed by the following process. As a typical p-type semiconductor, holes are the charge carriers in NiFe_2_O_4_. The evolution of absorbed oxygen into oxygen species, as confirmed by the above XPS analysis, will reduce the electrons in the sensing materials (Figure 9d). Therefore, the rich existence of holes generates a hole accumulation layer on the very surface of NiFe_2_O_4_, leading to a highly conductive state in the air. After exposure to a reductive gas atmosphere such as toluene, the reaction between these oxygen species and target gas molecules can release the trapped electrons back (Figure 9e), which results in the conductivity of the NiFe_2_O_4_ based sensor decreasing greatly. Thus, a high resistance state can obviously appear. For instance, a real resistance of about 82 kΩ was measured in the air for the NiFe_2_O_4_ based sensor, as shown in Figure 9f. After exposure to even 1 ppm toluene, the resistance dramatically increased to approximately 98 kΩ. When the NiFe_2_O_4_ based sensor was switched to air again, sufficient oxygen molecules alternatively adsorbed on the NiFe_2_O_4_ surface. As a result, the resistance restored its initial status, leading to the achievement of a whole response–recovery period. In contrast with the electrical conductivity seen in previous reports [61,62], our nano-octahedron NiFe_2_O_4_ exhibited good electrical conductivity with low resistivity. The variation ratio of resistance is heavily determined by the sensing materials and the concentrations of the target gas. The possible reactions are clarified in the following [1,63]

(1) $$O_{2}(\mathrm{gas})\to O_{2}(\mathrm{adsorbed})$$

(2) $$O_{2}({\mathrm{adsorbed})+e}^{-}\to O_{2}^{-}$$

(3) $$O_{2}^{-}{+e}^{-}\to 2O^{-}$$

(4) $$O^{-}{+e}^{-}\to O^{2-}$$

(5) $$C_{7}H_{8}{+9O}^{-}\to 7{\mathrm{CO}}_{2}{+4H}_{2}{O+9e}^{-}$$

(6) $$C_{7}H_{8}{+9O}^{2-}\;\to 7{\mathrm{CO}}_{2}{+4H}_{2}{O+18e}^{-}$$

In short, the unique assembly of NiFe_2_O_4_ nanoparticles into porous nano-octahedrons with hollow interiors can provide enough specific surface areas and space and rich active sites to catalyze toluene. The native features of NiFe_2_O_4_ may be suitable for the toluene catalyst at an optimum operating temperature due to the reversible redox reaction of ${\mathrm{Ni}}^{2+}↔{\mathrm{Ni}}^{3+},{\mathrm{Fe}}^{2+}↔{\mathrm{Fe}}^{3+}$[39]. Specifically, the as-prepared mesopore nano-octahedrons with hollow interiors easily facilitate the fast diffusion of gas molecules in reaching and departing from the sensing surface. In addition, a large number of NiFe_2_O_4_ may offer more active sites to boost the reaction between the target gas molecules and oxygen species. Thus, the as-synthesized NiFe_2_O_4_ nano-octahedrons show excellent sensing properties to low-ppm-level toluene. The response and selectivity could be further enhanced by proper doping, or appropriately coupling with other nanomaterials or functionalizing with noble metal nanoparticles. For instance, the Ni_0.33_Co_0.67_Fe_2_O_4_ microspheres showed the highest gas-sensing sensitivity to toluene due to their rich oxygen vacancies and large specific surface area [64]. Compared with individual α-Fe_2_O_3_ and NiFe_2_O_4_, the 1D α-Fe_2_O_3_/NiFe_2_O_4_ heterojunction showed better gas-sensing properties for acetone detection due to the enhanced charge separation and carrier4 depletion layer at the interface [39].

## 4. Conclusions

In summary, porous NiFe_2_O_4_ nano-octahedrons were successfully synthesized using a facile refluxing route via a simple and direct pyrolysis of NiFe-bimetallic MOFs. The cooperative effects of PTA and DAB play a critical role in the formation of octahedral NiFe-bimetallic MOFs precursors. The gas sensing performance of the NiFe_2_O_4_ based sensor was studied for toluene detection. As a result, the gas sensor exhibited the advantages of long-term stability (30 days), excellent repeatability, short response (25 s) and recovery (40 s) time, and a lower detection limitation (1 ppm). These resulting sensing properties may stem from the decent specific surface area (43.89) with a porous structure, hollow interiors, small size (30 nm), and the catalytic nature of NiFe_2_O_4_. More importantly, the easy fabrication strategy can be properly extended for the preparation of other well-defined micro-/nano-structures using both PTA and DAB as coordinated ligands.

## Figures and Tables

**Table 1:** Comparison of toluene sensing performance among the gas sensors based on typical p-type materials.

Materials	Microstructures	Concentration (ppm)	T (°C)	Response	Limit of Detection	Ref.
NiO	Flower-like	5	250	2.63	0.5 ppm	[48]
NiO	Nanoparticle	200	210	1.6	100 ppm	[49]
CuO	Flower	500	260	2.5	10 ppm	[50]
Co_3_O_4_	Nanorod	200	200	35	10 ppm	[51]
Co_3_O_4_	Nanosheet	100	150	6.08	1 ppm	[52]
Co_3_O_4_	Microsphere	100	180	2.2		[53]
Cr_2_O_3_	Microsphere	100	170	33.64	1 ppm	[54]
Ag-LaFeO_3_	Nanoparticle	5	215	24	5 ppm	[55]
NiGa_2_O_4_-NiO	Nanosphere	100	230	12.7	0.5 ppm	[56]
NiFe_2_O_4_	Ordered mesoporous	1	230	77.3	2 ppb	[57]
NiFe_2_O_4_	Hexagonal biyramid	200	140	5.73	5 ppm	[20]
NiFe_2_O_4_	Nano-octahedron	100	260	6.41	1 ppm	This work
